# Migraine and the Gut–Brain Axis—The Role of Microbiome-Targeted Biotics

**DOI:** 10.3390/nu18050720

**Published:** 2026-02-24

**Authors:** Márk Kozák, Tímea Sitku, Rebeka Hodossy-Takács, Flóra Sápi, István Várkonyi, Zsolt Barta

**Affiliations:** 1 Department of Infectology, Faculty of Medicine, University of Debrecen, Bartok Bela Street 2-26, 4031 Debrecen, Hungary; sitku.timea@med.unideb.hu (T.S.); varkonyi.istvan@med.unideb.hu (I.V.); barta@med.unideb.hu (Z.B.); 2 Division of Clinical Laboratory Sciences, Department of Laboratory Medicine, Faculty of Medicine, University of Debrecen, 4032 Debrecen, Hungary; hodossy.rebeka@med.unideb.hu; 3 MTA-DE Lendület “Momentum” Hemostasis and Stroke Research Group, 4032 Debrecen, Hungary; 4 Faculty of Medicine, University of Debrecen, 4032 Debrecen, Hungary; flora.sapi@gmail.com

**Keywords:** migraine, gut–brain axis, probiotics, prebiotics, postbiotics, microbiome, short-chain fatty acids, neuroinflammation

## Abstract

**Background:** Migraine is a highly prevalent and disabling primary headache disorder frequently accompanied by gastrointestinal symptoms and comorbid gastrointestinal diseases. Increasing evidence suggests that alterations in the gut microbiota and dysregulation of the microbiome–gut–brain axis may contribute to migraine pathophysiology through immune activation, oxidative stress, impaired intestinal barrier function, and neuroinflammatory signaling. **Objectives:** This narrative review aims to summarize current mechanistic and clinical evidence linking the gut–brain axis to migraine, with a particular focus on the potential roles of probiotics, prebiotics, and postbiotics as adjunctive strategies in migraine management. **Methods**: A narrative synthesis of experimental, translational, and clinical studies was performed, focusing on microbiome composition, gut barrier integrity, immune and oxidative pathways, and interventional trials evaluating probiotics, prebiotics, synbiotics, and microbiota-derived metabolites in adult and pediatric migraine populations. **Results**: Migraine has been associated with intestinal dysbiosis, increased gut permeability, and low-grade systemic inflammation. Probiotics, most commonly strains of *Lactobacillus* and *Bifidobacterium*, may modulate inflammatory cytokine profiles, enhance tight junction integrity, reduce oxidative stress, and influence neurotransmitter-related pathways along the gut–brain axis. Clinical trials evaluating probiotic supplementation report heterogeneous but promising signals, including reductions in migraine frequency, severity, disability scores, and analgesic use, particularly in chronic migraine and pediatric populations. Emerging evidence also supports a potential role for prebiotics (e.g., inulin-type fructans) and microbiota-derived metabolites such as short-chain fatty acids, although direct clinical data remain limited. **Conclusions**: Modulation of the microbiome–gut–brain axis represents a biologically plausible adjunct approach in migraine management. While probiotics, prebiotics, and postbiotics show potential benefits with favorable safety profiles, current evidence of their strain-, formulation-, and population-specific characteristics is lacking. Well-powered, placebo-controlled trials with standardized migraine endpoints and integrated microbiome and metabolomic analyses are needed to define responders, optimal interventions, and clinical relevance.

## 1. Introduction

Migraine is a prevalent primary headache disorder with significant morbidity and rising prevalence, affecting around 14% of the global population [1]. As a lifelong disorder with early onset beginning at approximately 7 years of age in boys and 10.9 years of age in girls [2], migraine is among the top causes of years lived with disability [3]. In addition to its high prevalence and disability burden, migraine is associated with substantial socioeconomic costs, with estimated direct and indirect expenditures of approximately USD 36 billion annually in the United States [4]. Clinically, migraine is characterized by recurrent attacks of moderate-to-severe headache lasting 4–72 h, typically unilateral and pulsating, aggravated by routine physical activity, and frequently accompanied by photophobia and phonophobia; in a substantial subset of patients, attacks are associated with reversible focal neurological symptoms called aura. Migraines can be categorized by the frequency of attacks: less than 15 headache days per month is considered episodic migraine, while 15 or more headache days per month for more than 3 months is considered chronic migraine [2,3,5].

Gastrointestinal (GI) symptoms and disorders are strongly interlinked with migraine. Over 70% of migraine patients exhibit some form of gastrointestinal disorder, and conversely, people with gastrointestinal disorders have a higher migraine prevalence [1].

The most common gastrointestinal disorders associated with migraine are irritable bowel syndrome (IBS) (reported in up to 50% of patients with migraine [6]), inflammatory bowel disease (IBD) [7,8,9], gastroesophageal reflux disease (GERD) [10], functional dyspepsia [11,12], cyclic vomiting syndrome [2], peptic ulcers and *Helicobacter pylori* infections [13,14]. Gastrointestinal symptoms may occur during the prodromal phase of migraine (in the form of food cravings) and are also common during an attack: nausea and vomiting are experienced by 60–95% and 50–62% of migraine patients, respectively, and may influence quality of life and therapeutic effectiveness [10]. An interesting phenomenon is the so-called abdominal migraine (AM). It is characterized by recurring episodes of abdominal pain accompanied by vasomotor symptoms. While it is more commonly observed in children, AM is rare and less well understood in adults. The diagnostic criteria for adult AM are not yet fully established, and treatment options, both pharmacological and non-pharmacological, have not been thoroughly evaluated in this population [15,16].

The gut–brain axis describes bidirectional communication between the gastrointestinal tract and the central nervous system through neural, immune, endocrine, and metabolic pathways, and the gut microbiota is increasingly recognized as an important modulator of this network [17]. The intestinal microbiome represents the largest and most complex microbial community in the human body, comprising an estimated 5000 microbial species and weighing approximately 2 kg [18]. It is dominated by bacteria but also includes archaea, viruses, and fungi [19]. Far from being a static ecosystem, the gut microbiota is highly dynamic and responsive to environmental influences, particularly to dietary factors. Functionally, it plays a central role in host metabolism and signaling by facilitating nutrient breakdown, synthesizing enzymes and vitamins (notably vitamin K), and producing bioactive metabolites and neurotransmitter-related compounds with potential neuromodulator effects [18,19]. This metabolic and signaling capacity, combined with its plasticity, provides a strong biological rationale for nutrition-based interventions aimed at modulating microbial composition and function, with potential downstream effects on systemic and neurological processes [18].

Migraine attacks are influenced by both non-dietary triggers, such as stress, sleep disturbance, and hormonal fluctuations, and dietary factors, including fasting, specific foods, and food additives. The frequent overlap between dietary triggers and gastrointestinal symptoms has driven growing interest in nutrition-based approaches as adjunctive strategies in migraine management, particularly when pharmacological options are insufficient or poorly tolerated [20].

Diet supplementation (with riboflavin, omega-3 fatty acids, alpha lipoic acid, magnesium, coenzyme Q10, ginger, and caffeine) has already been shown to have a beneficial effect on migraine patients [21].

Importantly, overall dietary patterns, particularly Mediterranean and low-glycemic index diets, strongly shape gut microbiome composition and function, influencing inflammatory and metabolic pathways relevant to migraine. This broader nutritional context supports microbiome-targeted interventions as lifestyle-linked adjuncts and underscores diet as a key confounder in biotic intervention trials [20].

Despite growing interest in nutrition-based modulation of migraine, evidence for the roles of prebiotics, probiotics, and postbiotics remains limited, prompting increasing interest in cause-oriented, lifestyle- and diet-based adjunctive approaches to complement pharmacotherapy in migraine prevention.

## 2. Materials and Methods

### 2.1. Study Design

This article is a narrative review focusing on the role of the microbiome–gut–brain axis in migraine, with particular emphasis on probiotics, prebiotics, and postbiotics as nutrition-based adjunctive strategies. The aim was to synthesize and contextualize mechanistic, translational, and clinical evidence rather than to perform a systematic review or meta-analysis.

### 2.2. Literature Identification and Selection

The relevant literature was identified through structured searches of major biomedical databases, including PubMed/MEDLINE, Scopus, and Web of Science, complemented by manual screening of reference lists from key review articles and relevant clinical trials. The search covered the period from database inception through December 2025, with the final search update performed on 15 January 2026.

Search strategies combined controlled vocabulary (where applicable) and free-text terms. Representative search combinations included (“migraine” OR “headache”) AND (“microbiome” OR “gut–brain axis” OR “intestinal permeability” OR “probiotics” OR “prebiotics” OR “postbiotics” OR “short-chain fatty acids” OR “dietary interventions”). Search strings were adapted as appropriate for each database.

In line with the narrative design of the review, formal inclusion and exclusion criteria and quantitative synthesis were not applied. Nevertheless, evidence selection followed predefined conceptual priorities. Preference was given to randomized controlled trials, placebo-controlled interventional studies, and high-quality systematic reviews. Mechanistic experimental studies were included when directly relevant to proposed microbiome–gut–brain pathways implicated in migraine pathophysiology. Study selection was guided by methodological rigor, clinical relevance, and contribution to conceptual synthesis.

Only peer-reviewed, English-language full-text publications were considered. This structured yet flexible approach was adopted to enhance transparency and reproducibility while preserving the integrative scope inherent to narrative synthesis.

### 2.3. Data Extraction and Synthesis

Data were extracted using a structured qualitative framework aligned with the predefined conceptual priorities of this review. Extraction focused on:

- Proposed biological mechanisms linking the gut microbiome to migraine pathophysiology;
- Characteristics of probiotic, prebiotic, synbiotic, or postbiotic interventions (e.g., Strain composition, formulation, duration, study population);
- Reported clinical outcomes (e.g., migraine frequency, severity, disability scores, analgesic use, quality-of-life measures);
- Methodological features and key limitations contributing to between-study heterogeneity.

Findings were synthesized thematically to integrate experimental, translational, and clinical perspectives. Particular attention was given to areas of convergence and divergence across trials, strain-specific effects, and potential sources of variability, including phenotype, dietary background, and outcome definition.

## 3. Results

### 3.1. The Gut–Brain Axis in Migraine

Migraines’ pathophysiology is complex and not yet fully understood. One possible pathogenic mechanism involves the imbalance of the synthesis and elimination of reactive oxygen species (ROS), causing oxidative stress. Patients with migraine seem to have an increased total oxidative status (TOS), along with nitric oxide (NO) and malondialdehyde (MDA), the latter of which is a useful marker of lipid peroxidation [3]. During a migraine attack, the levels of interleukins (e.g., IL-1 and IL-6), C-reactive protein (CRP) and tumor necrosis factor alpha (TNF-α) are increased [2]. These substances can increase cellular permeability and cell–cell interactions, leading to vascular dysfunction and subsequent neuropathic pain [21], and therefore, seem to be associated with attack severity [1]. Importantly, migraine is defined clinically based on symptom patterns rather than underlying etiology, allowing heterogeneous biological mechanisms to converge into a shared migraine phenotype [22]. Within this framework, activation of the trigeminovascular system is considered a core mechanism in migraine pathophysiology, as inflammatory mediators and oxidative stress lower the activation threshold of trigeminal nociceptors [23]. Pro-inflammatory cytokines and oxidative stress may further promote the release of migraine-relevant neuropeptides, particularly calcitonin gene-related peptide (CGRP), thereby amplifying neurogenic inflammation and pain transmission [24,25].

From a gut–brain axis perspective, peripheral immune and metabolic signals originating from the gastrointestinal tract may act as upstream modulators of established migraine-related pathways. Alterations in gut microbiome composition and intestinal barrier function may contribute to a chronic low-grade inflammatory state, facilitating sustained trigeminovascular sensitization [17]. Nociceptive signals conveyed by unmyelinated C fibers are relayed through the thalamus, which functions as a central sensory gatekeeper; sustained peripheral inflammatory input -potentially shaped by gut–brain signaling- may impair this gating, facilitating persistent pain perception and migraine chronification [17,26].

The human gut microbiome is dominated by several major bacterial taxa, most prominently *Bacteroidetes* and *Firmicutes*, with additional contributions from genera such as *Prevotella*, *Clostridium*, *Ruminococcus*, and *Eubacterium* [18]. The relative abundance of these phyla has been discussed in relation to host metabolism and immune signaling; however, phylum-level ratios (including *Firmicutes/Bacteroidetes*) show substantial variability across cohorts and conditions, and their interpretation as a universal marker of “health” or “dysbiosis” remains context-dependent. Accordingly, compositional shifts at this level should be interpreted cautiously rather than as disease-defining patterns [18].

Emerging evidence suggests that migraine may be associated with dysbiotic microbiome patterns. In children with migraine, reduced microbial diversity has been observed alongside an increased abundance of specific taxa, including *Eggerthella*, *Sutterella*, and *Eubacterium* species. Notably, *Eggerthella* enrichment has also been reported in other neuropsychiatric conditions, such as depression, schizophrenia, and bipolar disorder, supporting the concept of shared microbiome-linked neuroimmune pathways. These observations are cohort- and methodology-dependent and should be interpreted as associative rather than implying a consistent migraine-specific microbiome signature. In contrast, non-migraine controls tend to exhibit higher microbial diversity, underscoring a potential association between reduced microbiome diversity and migraine susceptibility [1].

Dysbiosis-related alterations in gut barrier integrity represent a plausible mechanistic bridge between the intestinal microbiome and migraine pathophysiology. Reduced expression of tight junction proteins, including claudin, occludin, and zonula occludens-1 (ZO-1), can increase intestinal permeability, facilitating translocation of lipopolysaccharides (LPS) derived from Gram-negative bacteria into the systemic circulation [18]. LPS acts as a potent endotoxin that activates Toll-like receptor 4 (TLR4), triggering downstream pro-inflammatory signaling and increased production of cytokines such as interleukins, TNF-α, and CRP [2].

Sustained activation of these inflammatory pathways may promote chronic low-grade systemic and neuroinflammation, enhance oxidative stress, and alter neurotransmitter and neuromodulator homeostasis, all of which are relevant to migraine biology [1,10]. In preclinical models, dysbiosis-associated increases in TNF-α within the caudal trigeminal nucleus have been linked to AMPA receptor (GluA1) phosphorylation and central nociceptive sensitization, providing a mechanistic bridge between gut-derived inflammation and migraine-like pain [27]. In parallel, microbiota-derived metabolites, including short-chain fatty acids (SCFAs), can modulate immune responses and neurotransmitter synthesis, while microbial production of amino acid precursors such as tryptophan, tyrosine, and glutamine contributes to the regulation of serotonergic, dopaminergic, and GABAergic signaling. Preclinical models suggest that selected SCFAs (e.g., butyrate and propionate) may influence neuroinflammatory signaling, including microglial activation states, through direct or indirect pathways involving blood–brain barrier transport; however, direct causal evidence in human migraine remains limited [20]. Importantly, not all microbiota-derived SCFAs exert uniformly beneficial effects. While acetate may show anti-inflammatory actions in the gut, experimental headache models implicate acetate in trigeminovascular sensitization, highlighting tissue- and context-dependent effects of microbial metabolites [10,28]. Consistent with this framework, migraine attacks are associated with transiently increased serotonin levels, whereas interictal periods are characterized by reduced serotonergic tone, highlighting a dynamic neurochemical interface between the gut microbiota and migraine expression [10].

### 3.2. Biological Mechanisms of Probiotics in Migraine

According to the World Health Organization/Food and Agriculture Organization (WHO/FAO) definition, probiotics are live microorganisms, including bacteria and fungi, which confer a health benefit on the host when administered in adequate amounts [3].

The majority of commercially available probiotic formulations contain strains belonging to the *Lactobacillus* and *Bifidobacterium* genera [29], while other preparations may include probiotic yeasts such as *Saccharomyces boulardii* [30]. The proposed beneficial effects of probiotics are primarily mediated through their interaction with the resident intestinal microbiota, as well as through immunomodulatory actions and attenuation of oxidative stress [3]. In addition, probiotics may influence migraine-relevant signaling by modulating tryptophan/serotonin pathways and downregulating pro-inflammatory cascades implicated in migraine, including MAPK- and NF-κB-related signaling [20].

Evidence from in vitro human intestinal model studies indicates that exposure to specific probiotic strains, including *Lactobacillus acidophilus*, *Lactobacillus plantarum*, *Lactobacillus rhamnosus*, and *Enterococcus faecium,* is associated with an increased production of anti-inflammatory cytokines (notably IL-10 and IL-6), accompanied by a reduction in pro-inflammatory mediators, such as IL-8, CXCL-10, and MCP-1. In parallel, an increase in SCFA production, predominantly butyrate, has been observed, with measurable effects emerging after approximately three weeks of probiotic administration [31].

Findings from in vivo studies further support strain- and context-specific immunomodulatory effects. Administration of *Lactobacillus rhamnosus* in combination with citrus fibers resulted in a significant reduction in serum CRP and TNF-α levels after four weeks, whereas supplementation with *Bifidobacterium lactis* alone for a similar duration did not significantly alter CRP or cytokine concentrations in healthy individuals [31].

These observations underscore the importance of probiotic strain selection, co-administered substrates, and host characteristics when interpreting immunological outcomes.

Beyond immune modulation, probiotics may influence migraine-relevant pathways through effects on intestinal barrier integrity. Tight junction proteins are regulated, in part, by bacterial stimulation of TLRs, which can promote the expression and stabilization of claudin, occludin, and zonula occludens proteins. Experimental data suggest that supplementation with *Lactobacillus plantarum* and *Lactobacillus acidophilus* increases occludin expression [32], while *Lactobacillus plantarum* has also been shown to enhance ZO-1 expression in the duodenum [33]. In addition, *Bifidobacterium* supplementation appears to preserve the localization of occludin and claudin at tight junctions, thereby supporting barrier integrity [32].

**Figure 1:**
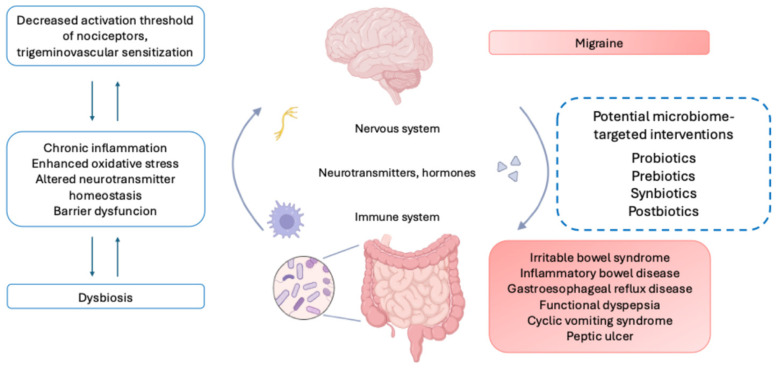
Conceptual overview of microbiome–gut–brain axis mechanisms in migraine and potential intervention targets.

Taken together, these findings support the conceptualization of migraine as a systems-level disorder in which gut barrier integrity, immune and oxidative signaling, and microbiota-derived metabolites interact to shape nociceptive processing along the gut–brain axis (Table 1 and Figure 1). This integrated mechanistic framework provides a coherent biological rationale for the use of probiotics in migraine, while also helping to explain the heterogeneity of clinical trial outcomes, which likely reflects differences in strain composition, host microbiome background, dietary context, and migraine phenotype.

Schematic overview of proposed microbiome–gut–brain axis interactions in migraine. Alterations in gut microbiome composition and gastrointestinal function may impair intestinal barrier integrity and promote immune activation, oxidative stress, and alter neurotransmitter-related signaling. These processes can lower the activation threshold of trigeminal nociceptors, facilitating trigeminovascular sensitization and migraine expression. Microbiota-derived metabolites, including short-chain fatty acids, are highlighted as candidate modulators linking intestinal microbial activity to immune and neural pathways. Potential microbiome-targeted interventions (probiotics, prebiotics, synbiotics, and postbiotics) are depicted as upstream modulators of established migraine mechanisms rather than independent causal drivers.

### 3.3. Clinical Evidence for Microbiome-Targeted Interventions in Migraine

#### 3.3.1. Probiotics: Adult Clinical Trial Evidence

Early studies showed feasibility of administering probiotics to migraine patients. A 2015 open-label pilot study concluded that a multi-species probiotic (*Bifidobacterium bifidum*, *Bifidobacterium lactis*, *Lactobacillus acidophilus*, *Lactobacillus brevis*, *Lactobacillus casei*, *Lactobacillus salivarius*, and *Lactobacillus lactis*) administered for 12 weeks resulted in a decrease in migraine days. The participants of this study were adult migraine patients with at least 4 attacks per month. The study excluded chronic migraine patients [34].

However, subsequent placebo-controlled randomized trials yielded mixed results. In 2017, the 2015 open-label study was replicated as a randomized placebo-controlled study. The study used a similar multi-strain probiotic (*Bifidobacterium bifidum*, *Bifidobacterium lactis*, *Lactobacillus acidophilus*, *Lactobacillus brevis*, *Lactobacillus casei*, *Lactobacillus salivarius*, *Lactobacillus lactis*), which had to be taken daily for 12 weeks. The subjects met the same criteria as in the previous study, and chronic migraine patients were again excluded. The subjects met the same criteria as in the previous study, and chronic migraine patients were again excluded. With this new study, no significant change was found in migraine frequency, intensity, or drug usage. Nevertheless, patients receiving probiotics reported greater improvement in quality of life compared to the placebo group [35].

In contrast to these neutral findings, a randomized, double-blind, placebo-controlled 2019 study using a 14-strain multi-species probiotic for over 10 weeks in episodic migraine patients and 8 weeks in chronic migraine patients showed significantly reduced attack frequency and severity in both types of migraine, with a higher benefit observed in chronic migraine cases [36].

More recently, attention has shifted toward combination strategies. A parallel randomized, triple-blind, placebo-controlled 2024 study, conducted on 72 adult patients, examined the effect of probiotics co-administered with Vitamin D supplementation in migraine therapy [2]. The probiotic supplement used in the study contained multiple species of Lactobacillus (*Lactobacillus plantarum*, *Lactobacillus casei*, *Lactobacillus acidophilus*, *Lactobacillus bulgaricus*) and *Bifidobacterium* (*Bifidobacterium infantis*, *Bifidobacterium longum*, and *Bifidobacterium breve*), and *Streptococcus thermophilus*, and it was administered with Vitamin D for 12 weeks. The study showed a significant reduction in headache frequency; a borderline reduction in severity was reported, whereas headache duration was not significantly different between groups [2]. Patients’ serum contained decreased levels of NO and MDA, and TOS was also reduced [3].

In adult populations, phenotype-specific effects have also been reported. In a large randomized, placebo-controlled trial including 247 patients with vestibular migraine, four months of supplementation with *Lactobacillus casei Shirota* was associated with significantly milder symptoms compared with placebo, suggesting that probiotic efficacy may vary across migraine subtypes and clinical phenotypes [37].

#### 3.3.2. Pediatric Probiotic Studies and Adjunctive Use

Pediatric data, although more limited, provide additional support for adjunctive probiotic strategies.

A clinical trial was conducted in 2024 on 41 children aged 5–15, comparing two groups: one received propranolol with probiotics, and the other received propranolol with a placebo. The probiotic used contained *Saccharomyces cerevisiae*. A statistically significantly higher decrease in the number of attacks per month and in disability scores (measured by Pediatric Migraine Disability Assessment Scale [PedMIDAS]) was observed in the intervention group, compared to the control group [29].

Similar findings were observed with other prophylactic combinations. Probiotic and sodium valproate co-administration was also tested in a double-blind, randomized controlled 2023 clinical trial. The 80 patients were children aged 6–15 years. The multi-strain probiotic *(Lactobacillus acidophilus*, *Lactobacillus rhamnosus*, *Lactobacillus bulgaricus*, *Lactobacillus casei*, *Bifidobacterium breve*, *Bifidobacterium infantis* and *Streptococcus thermophilus*) was administered for 4 months. The trial found a significant improvement in headache severity and frequency and reduced daily painkiller consumption compared to placebo [38].

#### 3.3.3. Prebiotics and Synbiotics

According to the International Scientific Association for Probiotics and Prebiotics (ISAPP) consensus, prebiotics are non-digestible substrates that are selectively utilized by host microorganisms, typically dietary fibers, thereby nourishing beneficial microbes and conferring a health benefit to the host, analogous to a fertilizer effect on the intestinal microbiota [39].

Within the migraine–microbiome framework, the rationale for prebiotics is primarily mechanistic. By enriching SCFA-producing commensals and increasing fermentation products such as butyrate and propionate, prebiotics may strengthen intestinal barrier function, reduce systemic inflammatory tone, and attenuate trigeminovascular sensitization along the gut–brain axis. By selectively promoting *Bifidobacterium* growth, prebiotics may additionally reduce endotoxemia through decreased lipopolysaccharide translocation, thereby attenuating TNF-α-driven inflammatory signaling relevant to migraine pathophysiology [4,40].

Although clinical evidence remains limited, a randomized, double-blind, placebo-controlled trial in women demonstrated that 10 g/day of inulin administered for 12 weeks improved migraine symptoms and mental health indices, supporting the potential role of prebiotics in migraine prophylaxis [41,42].

In addition to prebiotics alone, a related body of interventional work has used synbiotics (probiotics plus a prebiotic such as fructooligosaccharides), showing improvements in migraine characteristics and inflammatory markers, although the specific contribution of the prebiotic component cannot be isolated in these designs [4].

Population-level observations are consistent with the same direction of effect. Higher dietary fiber intake (a key driver of colonic fermentation and SCFA generation) has been associated with a lower prevalence of severe headache/migraine, including an estimated reduction per 10 g/day higher fiber intake, though causality cannot be inferred from cross-sectional analyses [43]. Overall, prebiotics, especially inulin-type fructans, represent a promising, biologically plausible adjunct in migraine, but the evidence base remains small, largely sex- and setting-specific, and would benefit from independent replication with standardized migraine endpoints and integrated microbiome/metabolite readouts [41].

#### 3.3.4. Postbiotics

Postbiotics are defined as a preparation of inanimate microorganisms and/or their components that confers a health benefit on the host [44]. In this framework, postbiotics typically include non-viable (killed/inactivated) microbial cells and/or structural cell components (e.g., cell wall fragments, surface proteins, and exopolysaccharides), and they may contain metabolites generated during production; however, purified microbial metabolites alone (e.g., isolated organic acids) are not, strictly speaking, postbiotics under the ISAPP definition.

#### 3.3.5. Microbiota-Derived Metabolites (SCFAs)

In the migraine–microbiome literature, the term is often used more broadly to denote microbiota-derived bioactive metabolites that could mediate gut–brain effects, most prominently SCFAs, such as butyrate and propionate [45]. Mechanistically, SCFAs are plausible modulators of migraine biology because they can influence intestinal barrier function, systemic immunity, and neuroinflammatory signaling along the gut–brain axis, all of which intersect with trigeminovascular sensitization and attack susceptibility [46]. Nevertheless, the strongest evidence base for direct SCFA administration currently derives from preclinical models rather than human trials. Preclinical data provide some of the most direct support for this concept. In nitroglycerin-induced migraine mouse models, exogenous sodium butyrate and sodium propionate attenuated migraine-like hyperalgesia/photophobia, reduced inflammatory readouts, and were reported to partly normalize gut pathology and microbiota composition [46].

Taken together, these studies suggest that restoring SCFA signaling, whether via diet/prebiotics, live biotics that increase endogenous SCFA production, or metabolite-focused interventions, may represent a biologically grounded adjunct strategy. Nonetheless, human evidence for defined postbiotic products and for metabolite-focused interventions in migraine remains limited, and translation will require well-powered, formulation-specific clinical trials with standardized migraine endpoints.

#### 3.3.6. Integrated Interpretation

Across the studies summarized, clinical signals are most consistent for selected probiotic and synbiotic formulations and inulin-type prebiotics, but results remain heterogeneous, supporting the need for strain- and formulation-specific, adequately powered trials with standardized migraine endpoints and integrated microbiome/metabolite readouts (Table 2).

## 4. Discussion

Taken together, the available mechanistic and clinical evidence supports the microbiome–gut–brain axis as a biologically plausible and generally low-risk adjunct target in migraine management. This concept appears particularly relevant in patients with prominent gastrointestinal symptoms, comorbid gastrointestinal disease, or suboptimal tolerance or response to standard preventive therapies. The frequent coexistence of migraine with disorders such as IBS, IBD, GERD, and functional dyspepsia reinforces the view that peripheral enteric immune–metabolic signaling may meaningfully influence trigeminovascular excitability and central sensitization [1,7,8,9,10,20,24].

**Figure 2:**
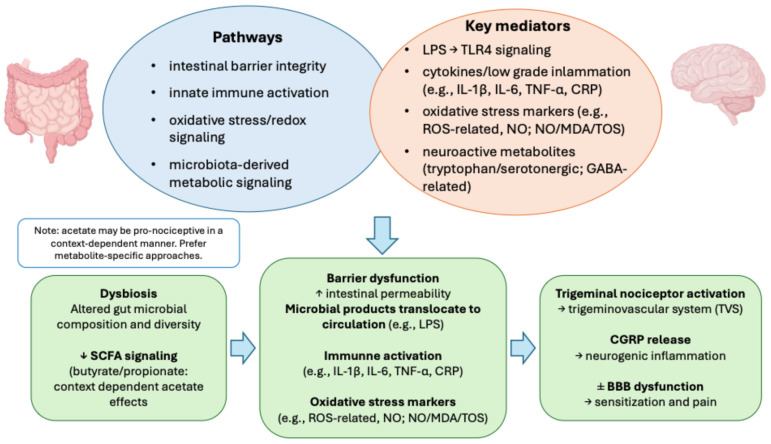
Proposed mechanisms linking gut dysbiosis to migraine. Abbreviations. BBB: blood–brain barrier; CGRP: calcitonin gene-related peptide; CRP: C-reactive protein; GABA: gamma-aminobutyric acid; IL: interleukin; LPS: lipopolysaccharide; MDA: malondialdehyde; NO: nitric oxide; ROS: reactive oxygen species; SCFA: short-chain fatty acid; TLR4: toll-like receptor 4; TNFα: tumor necrosis factor alpha; TOS: total oxidant status; TVS: trigeminovascular system. Symbols: ↑ increase; ↓ decrease; ± variable or context-dependent effect.

Rather than representing a novel or independent migraine mechanism, gut–brain axis alterations likely function as upstream modulators of established migraine biology. As outlined in earlier sections, disturbances in intestinal barrier integrity, innate immune activation, oxidative stress, and microbiota-derived metabolic signaling converge on core migraine pathways, including trigeminovascular activation and CGRP-mediated neurogenic inflammation (Figure 2). Importantly, the symptom-based clinical definition of migraine allows heterogeneous biological inputs to converge into a shared phenotype, providing a conceptual framework for understanding why microbiome-targeted interventions may yield variable and context-dependent effects across trials [23].

Gut dysbiosis may reduce beneficial microbial signaling, notably short-chain fatty acids (SCFAs), and impair epithelial barrier integrity, leading to increased intestinal permeability and systemic exposure to microbial products such as lipopolysaccharide (LPS). Subsequent innate immune activation (e.g., TLR4 signaling), cytokine production, and oxidative stress may function as upstream modulators converging on established migraine biology, including trigeminovascular activation and calcitonin gene-related peptide (CGRP)-mediated neurogenic inflammation, with additional contributions from blood–brain barrier dysfunction and neuroactive metabolic signaling.

Observational microbiome studies consistently suggest an association between migraine and altered microbial diversity or composition; however, the absence of a reproducible, disease-specific microbiome signature limits causal inference. Variability in sequencing platforms, analytical pipelines, dietary exposure, medication use, and comorbidities likely contributes substantially to between-study heterogeneity. These limitations underscore the need to interpret microbiome findings as associative and modulatory, rather than deterministic, within the broader migraine pathophysiological landscape [10,18,19,20].

Clinical interventional studies mirror this complexity. While several probiotic and synbiotic trials report improvements in migraine frequency, severity, disability, or analgesic consumption, other randomized placebo-controlled studies demonstrate neutral effects on primary endpoints, occasionally with improvements confined to quality-of-life measures. Collectively, these data argue against class-level efficacy and instead support strain-, formulation-, and phenotype-specific effects, as emphasized in recent systematic syntheses [10,34,35,36]. Pediatric studies often conducted in adjunctive designs are encouraging but inherently limit isolation of independent probiotic effects, highlighting the need for carefully designed monotherapy trials [29,38]. Evidence for prebiotics remains preliminary but biologically coherent, with inulin-type fructans showing signal in controlled trials that warrants replication using standardized migraine outcomes [41].

An important translational consideration emerging from metabolite-focused research is that microbiota-derived signals are not uniformly beneficial. Although butyrate and propionate are frequently framed as protective through barrier-supporting and immunomodulatory actions, experimental data indicate that acetate may exert context-dependent pro-nociceptive effects, including facilitation of trigeminovascular sensitization. This nuance cautions against non-specific strategies aimed at globally increasing SCFA levels and instead supports metabolite-specific and mechanism-informed approaches, particularly when considering postbiotic or metabolite-based interventions [10,45,46].

**Evidence quality and sources of heterogeneity.** Several shared design features likely contribute to mixed outcomes across trials: relatively small samples, short follow-up periods, variability in endpoints (frequency, severity, disability indices), heterogeneity of migraine phenotype (episodic vs. chronic; vestibular subtype), and limited control for baseline diet, GI comorbidities, and concurrent medication exposure. Importantly, pediatric studies are frequently adjunctive (probiotics added to propranolol or valproate), which limits isolation of an independent probiotic effect [29,38]. Similarly, co-supplementation designs (e.g., probiotic plus vitamin D) complicate attribution of clinical effects to a single intervention component [2,3]. In aggregate, the strength of evidence appears highest for selected probiotic formulations (strain- and context-specific), lower for synbiotics and prebiotics due to fewer trials, and currently largely preclinical for defined postbiotic and metabolite-focused interventions [10,41,46].

**Figure 3:**
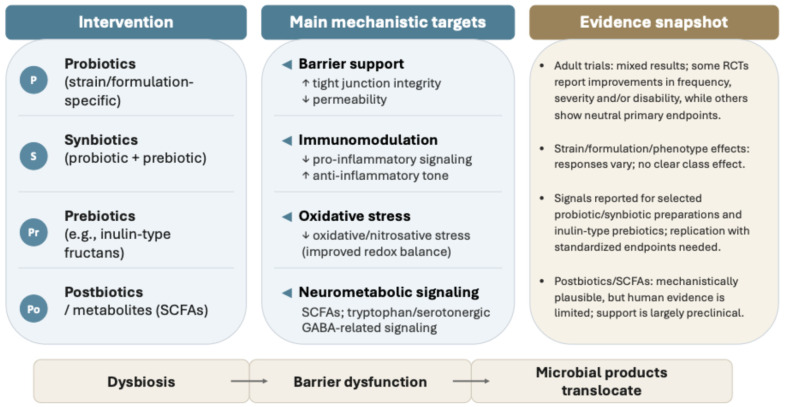
Microbiome-targeted adjunct strategies in migraine: mechanisms and evidence. Abbreviations: GABA: gamma-aminobutyric acid; RCT: randomized controlled trial; SCFA: short-chain fatty acid. Symbols: ↑ increase; ↓ decrease.

Overall, the most consistent clinical signals currently support selected probiotic and synbiotic formulations and, more tentatively, inulin-type prebiotics, while defined postbiotic strategies remain largely preclinical. Future studies should prioritize standardized migraine endpoints, strain- and formulation-specific designs, rigorous control of dietary and pharmacological confounders, and integrated microbiome–metabolomic–barrier biomarker analyses. Such approaches will be essential to identify responder subgroups, link clinical effects to underlying mechanisms, and clarify the role of microbiome-targeted strategies within precision-oriented migraine care [10,18,20,31,32] (Figure 3).

**Standard migraine therapies and potential gut-related considerations.** In routine practice, acute migraine treatment commonly involves non-steroidal anti-inflammatory drugs (NSAIDs), triptans and CGRP receptor antagonists (gepants), and preventive options include beta-blockers, antiepileptics, antidepressants, onabotulinumtoxin A (for chronic migraine), calcium channel blockers (e.g., flunarizine), angiotensin receptor blockers (e.g., candesartan), and CGRP-pathway-targeting agents [47,48]. From a gut–brain axis perspective, several of these drugs may influence gastrointestinal physiology, including nausea, delayed gastric emptying, constipation or diarrhea, and mucosal irritation. NSAIDs are associated with dyspepsia, gastritis, and increased gastrointestinal bleeding risk, and experimental data suggest that they may alter gut barrier integrity and microbiota composition [47,49,50]. Triptans may induce nausea and delayed gastric emptying but have no demonstrated direct microbiome effects [49,50]. Among preventive therapies, beta-blockers and angiotensin receptor blockers are generally well tolerated with rare gastrointestinal adverse events. Antiepileptic drugs (e.g., topiramate and valproate) and tricyclic antidepressants (e.g., amitriptyline) may cause constipation, diarrhea, or weight changes, potentially interacting with gut–brain signaling pathways. Flunarizine may be associated with constipation and weight gain [47,48,49,50]. OnabotulinumtoxinA does not exert clinically meaningful systemic gastrointestinal effects. CGRP monoclonal antibodies and gepants are generally well tolerated, with constipation being the most frequently reported gastrointestinal adverse event, particularly with receptor-targeting therapies [47,49,50,51].

Overall, although most standard migraine therapies are not expected to directly modify the gut microbiome, their effects on gastrointestinal physiology and tolerability may influence the broader gut–brain environment. Background pharmacotherapy should therefore be considered when evaluating microbiome-targeted adjunct strategies.

Future trials should therefore integrate standardized migraine outcomes with careful control of diet and medication exposure and mechanistic microbiome–metabolomic readouts to identify clinically meaningful responder profiles.

Probiotics, synbiotics, and prebiotics may influence migraine susceptibility through effects on intestinal barrier integrity, immunomodulation, redox and oxidative stress pathways, and microbiota-derived metabolic signaling, including short-chain fatty acids and serotonin- and GABA-related pathways. Clinical findings are heterogeneous and support strain-, formulation-, and phenotype-specific effects rather than class-level efficacy; pediatric studies are frequently adjunctive. Defined postbiotic or metabolite-based strategies remain largely preclinical, and clinical translation will require adequately powered trials with standardized migraine endpoints and integrated microbiome, metabolomic, and barrier-related biomarker readouts.

## Figures and Tables

**Table 1:** Microbiome–gut–brain axis mechanisms in migraine and conceptual biotic intervention targets.

Domain	What is Proposed/Observed in Migraine (as Summarized in the Review)	Key Mediators Highlighted	Potential “Biotics” Leverage Point (Conceptual)
Microbiome ecology	Dysbiosis and reduced functional resilience associated with attack susceptibility	Altered taxa/functional output	Rebalance community structure; restore functional metabolite production
Intestinal barrier	Increased permeability may facilitate systemic immune activation	Tight-junction regulation (e.g., occludin, claudin, ZO-1)	Support barrier integrity; reduce translocation of pro-inflammatory signals
Innate immune signaling	Microbial products may trigger inflammatory cascades	LPS → TLR4 → cytokines (e.g., IL-1, IL-6, TNF-α), CRP	Decrease inflammatory tone via microbial modulation and barrier effects
Neuroinflammation and sensitization	Low-grade inflammation may facilitate trigeminovascular sensitization	Pro-inflammatory cytokines; neuroimmune crosstalk and central nociceptive sensitization	Immunomodulation → reduced central/peripheral sensitization
Oxidative/nitrosative stress	Oxidative stress markers are discussed as modifiable correlates	NO, MDA, TOS, TAC	Reduce oxidative stress directly or indirectly via reduced inflammation
Neurotransmitter- related pathways	Microbial metabolism may influence precursor availability and neuromodulation	Tryptophan/serotonin-related pathways; other neuromodulators	Modulate precursor metabolism and gut–brain signaling
Microbial metabolites	SCFAs are presented as key mechanistic candidates	SCFAs (e.g., butyrate/ propionate)	Increase endogenous SCFA production (prebiotics/ probiotics); metabolite- focused approaches

Abbreviations: CRP: C-reactive protein; IL: Interleukin; LPS: Lipopolysaccharide; MDA: Malondialdehyde; NO: Nitric oxide; SCFAs: Short-chain fatty acids; TAC: Total antioxidant capacity; TLR4: Toll-like receptor 4; TOS: Total oxidant status; ZO-1: Zonula occludens-1.

**Table 2:** Interventional evidence summarized in the review: probiotics, prebiotics, and postbiotic/metabolite concepts in migraine.

Evidence Category	Key Study (Author, Year)	Population	Intervention (as Described in the Review)	Duration (Reported)	Main Findings Reported	Key Limitations Noted/Implicit
Adult RCT (mixed)	De Roos et al., 2015; De Roos et al., 2017 [34,35]	Adults with episodic migraine	Multispecies probiotic vs. placebo	12 weeks	Inconsistent effects on frequency/ severity; QoL improvement reported in some studies	Neutral primary outcomes; heterogeneous endpoints; chronic migraine excluded
Adult RCT (mixed)	Martami et al., 2019 [36]	Adults with episodic and chronic migraine	14-strain multispecies probiotic vs. placebo	10 weeks (episodic); 8 weeks (chronic)	Significant reduction in attack frequency and severity, more pronounced in chronic migraine	Formulation-specific; relatively short duration
Adult RCT with biomarker focus	Tirani et al., 2024; Tirani et al., 2025 [2,3]	Adults with migraine	Multispecies probiotic + vitamin D vs. placebo; oxidative stress, inflammatory biomarkers assessed	12 weeks	Reduction in migraine frequency; modest effects on severity/duration; decreased NO, MDA, and TOS	Combination design limits attribution to probiotic alone; biomarker changes may not map directly to clinical endpoints
Adult phenotype-specific RCT	Qi et al., 2020 [37]	Adults with vestibular migraine	Lactobacillus casei Shirota vs. placebo	4 months	Significantly milder vestibular migraine symptoms compared with placebo	Phenotype-specific population; strain-specific effect; limited generalizability to typical migraine
Pediatric adjunct RCT	Bazmamoum et al., 2024 [29]	Children (5–15 years) with migraine	Probiotic + propranolol vs. placebo + propranolol	As reported	Greater reduction in attack frequency and PedMIDAS disability	Adjunct design limits isolation of probiotic effect; pediatric-only data
Pediatric adjunct RCT	Bidabadi et al., 2023 [38]	Children (6–15 years) with migraine	Multispecies probiotic + sodium valproate vs. placebo + sodium valproate	~4 months	Improved headache severity/ frequency; reduced daily analgesic use	Adjunct design; formulation-specific; long-term durability unclear
Prebiotic- specific RCT signal	Vajdi et al., 2024; Vajdi et al., 2023 [41,42]	Adults (female cohort emphasized)	Inulin-type prebiotic (10 g/day) vs. placebo	12 weeks	Improvement in migraine indices and mental health measures; improvements in selected inflammatory and oxidative stress biomarkers (NO, TAC, hs-CRP); no change in (TOS).	Single study; sex- and setting-specific; replication needed
Synbiotic trials	Ghavami et al., 2021 [4]	Adults with migraine	Probiotic + prebiotic (e.g., FOS)	As reported	Improvements in migraine characteristics and inflammatory biomarkers	Contribution of Prebiotic component cannot be disentangled
Postbiotic/ metabolite concept	Lanza et al., 2021 [46]	Preclinical (mouse model)	SCFA administration (butyrate and propionate)	—	Attenuation of migraine-like behaviors and inflammatory readouts	Preclinical evidence only; human translation uncertain

Abbreviations: FOS: Fructooligosaccharides; MDA: Malondialdehyde; NO: Nitric oxide; PedMIDAS: Pediatric Migraine Disability Assessment Scale; QoL: Quality of life; RCT: Randomized controlled trial; SCFA: Short-chain fatty acid; TOS: Total oxidant status.

## Data Availability

This manuscript is a narrative review synthesizing previously published literature and does not generate new primary datasets.

## Abbreviations

The following abbreviations are used in this manuscript:

AM	abdominal migraine
CGRP	calcitonin-gene related peptide
CRP	C-reactive protein
FAO	Food and Agriculture Organization
GERD	gastroesophageal reflux disease
IBD	inflammatory bowel disease
IBS	irritable bowel syndrome
IL	interleukin
ISAPP	International Scientific Association for Probiotics and Prebiotics
LPS	lipopolysaccharide
MDA	malondialdehyde
NO	nitric oxide
PedMIDAS	Pediatric Migraine Disability Assessment Scale
QoL	quality of life
ROS	reactive oxygen species
SCFA	short-chain fatty acid
TAC	total antioxidant capacity
TLR	toll-like receptor
TNF-α	tumor necrosis factor alpha
TOS	total oxidative status
WHO	World Health Organization
ZO-1	zonula occludens-1
